# Maternal pregnancy hormone levels in an area with a high incidence (Boston, USA) and in an area with a low incidence (Shanghai, China) of breast cancer

**DOI:** 10.1038/sj.bjc.6690003

**Published:** 1999-09-24

**Authors:** L Lipworth, C-Ce Hsieh, L Wide, A Ekbom, S-Z Yu, G-P Yu, B Xu, S Hellerstein, K Carlstrom, D Trichopoulos, H-O Adami

**Affiliations:** Department of Epidemiology and Center for Cancer Prevention, Harvard School of Public Health, Boston, MA 02115, USA; Department of Medical Epidemiology Karolinska Institutet, 77 Stockholm S-171, Sweden; Cancer Center University of Massachusetts Medical Center, Worcester, MA 01605, USA; Department of Clinical Chemistry University Hospital, Uppsala S-751 85, Sweden; Department of Epidemiology School of Public Health, Shanghai Medical University, Shanghai, China; Department of Obstetrics and Gynecology Beth Israel Hospital, Boston, MA 02115, USA; Department of Obstetrics and Gynecology Huddinge Hospital, Stockholm S-17177, Sweden

**Keywords:** epidemiology, pregnancy steroids, breast cancer

## Abstract

Characteristics probably associated with the fetal hormonal milieu have recently been shown to increase (birth size indicators, prematurity, neonatal jaundice) or decrease (pregnancy toxaemia) breast cancer risk in the female offspring. However, it is unknown whether differences in pregnancy hormone levels may contribute to the marked geographical variation in breast cancer incidence. We have compared, in a highly standardized manner, pregnancy hormone levels in a population with high incidence and one with low incidence of breast cancer. Three hundred and four pregnant Caucasian women in Boston and 334 pregnant Chinese women in Shanghai were enrolled from March 1994 to October 1995. Levels of oestradiol, oestriol, prolactin, progesterone, human growth hormone, albumin and sex hormone-binding globulin were measured in maternal blood at weeks 16 and 27 of gestation and compared between the two study sites using non-parametric Wilcoxon's rank-sum test. Demographical, anthropometrical and pregnancy characteristics were ascertained through interview, and relevant variables concerning delivery and the newborn were abstracted from medical records and paediatric charts. During the first visit, median serum levels of all studied hormones were statistically significant, and in most instances substantially, higher among Chinese women, who have a low incidence of breast cancer, compared with American women, who have a high incidence of breast cancer. An analogous pattern was evident during the second visit, although the relative differences tended to be smaller. Further research is needed to identify lifestyle or other exogenous determinants of pregnancy hormone levels, as well as possible mechanisms by which they may influence carcinogenic processes in the breast and possibly other organs. © 1999 Cancer Research Campaign


British Journal of Cancer (1999) 79(1), 7–12
© 1999 Cancer Research Campaign

### Maternal pregnancy hormone levels in an area with a high incidence (Boston, USA) and in an area with a low incidence (Shanghai, China) of breast cancer

**L Lipworth[1,2], C-c Hsieh[1,3], L Wide[4], A Ekbom[1,2], S-z Yu[5], G-p Yu[5], B Xu[5], S Hellerstein[6], K Carlstrom[7], D Trichopoulos[1] and H-O Adami[1,2]**

[1]Department of Epidemiology and Center for Cancer Prevention, Harvard School of Public Health, 677 Huntington Avenue, Boston, MA 02115, USA; [2]Department of Medical Epidemiology, Karolinska Institutet, S-171 77 Stockholm, Sweden; [3]Cancer Center, University of Massachusetts Medical Center, 373 Plantation Street Suite 202, Worcester, MA 01605, USA; [4]Department of Clinical Chemistry, University Hospital, S-751 85 Uppsala, Sweden; [5]Department of Epidemiology, School of Public Health, Shanghai Medical University, Shanghai, China; [6]Department of Obstetrics and Gynecology, Beth Israel Hospital, Boston, MA 02115, USA; [7]Department of Obstetrics and Gynecology, Huddinge Hospital, S-171 77 Stockholm, Sweden

**Summary** Characteristics probably associated with the fetal hormonal milieu have recently been shown to increase (birth size indicators, prematurity, neonatal jaundice) or decrease (pregnancy toxaemia) breast cancer risk in the female offspring. However, it is unknown whether differences in pregnancy hormone levels may contribute to the marked geographical variation in breast cancer incidence. We have compared, in a highly standardized manner, pregnancy hormone levels in a population with high incidence and one with low incidence of breast cancer. Three hundred and four pregnant Caucasian women in Boston and 334 pregnant Chinese women in Shanghai were enrolled from March 1994 to October 1995. Levels of oestradiol, oestriol, prolactin, progesterone, human growth hormone, albumin and sex hormone-binding globulin were measured in maternal blood at weeks 16 and 27 of gestation and compared between the two study sites using non-parametric Wilcoxon's rank-sum test. Demographical, anthropometrical and pregnancy characteristics were ascertained through interview, and relevant variables concerning delivery and the newborn were abstracted from medical records and paediatric charts. During the first visit, median serum levels of all studied hormones were statistically significant, and in most instances substantially, higher among Chinese women, who have a low incidence of breast cancer, compared with American women, who have a high incidence of breast cancer. An analogous pattern was evident during the second visit, although the relative differences tended to be smaller. Further research is needed to identify lifestyle or other exogenous determinants of pregnancy hormone levels, as well as possible mechanisms by which they may influence carcinogenic processes in the breast and possibly other organs.

**Keywords:** epidemiology; pregnancy steroids; breast cancer

One of the challenging observations in breast cancer epidemiology is the remarkable international variability of the disease (Lipworth, 1995), Caucasian women in the US and UK having substantially higher incidence than Asian women in China and Japan despite rising rates among the latter. This variability cannot be accounted for by genetic factors because the incidence of breast cancer among the descendants of migrants from low-risk to high-risk countries eventually reaches the incidence prevailing in the host country (Ziegler et al, 1993). Although models have been proposed to accommodate the international variation in breast cancer occurrence on the basis of known risk factors (Pike et al, 1983), it appears that a substantial portion of this variation between countries cannot be accounted for by the established risk factors for breast cancer (Hsieh et al, 1990). Furthermore, most analytical epidemiological studies exploring the role of diet during adult life in the aetiology of breast cancer have found, at most, weak associations; the relative risks linking any particular nutrient or dietary pattern to this disease have been too close to the null value of 1 to explain the large interpopulation variation (Rogers and Longnecker, 1988; Hunter et al, 1996). It appears, therefore, that the studied conditions or events during a woman's adult life have not provided an adequate explanation of breast cancer occurrence patterns.

The idea that breast cancer originates during early life was advanced by MacMahon and colleagues on the basis of findings concerning the striking protective effect of an early first full-term pregnancy (Cole and MacMahon, 1969). Others have made similar suggestions on the basis of the association between adult body height and breast cancer risk (deWaard, 1975; Adami et al, 1990), an association which is evident in international correlations and also has been found in a number of case–control (Valaoras et al, 1969; Hsieh et al, 1990) and cohort (Tretli, 1989; van den Brandt et al, 1997) studies. In addition, the gradual increase of breast cancer risk over several generations of migrants points to early events and conditions that could also include transgeneration components (Ziegler et al, 1993). This last view is compatible with experimental evidence in laboratory animals in which prenatal exposures have been shown to increase the risk of cancer in the offspring of several species (Tomatis, 1989).

*Received 12 February*
*Revised 18 May 1998*
*Accepted 18 June 1998*

*Correspondence to:* L Lipworth, (present address) Department of Preventive Medicine, Vanderbilt University School of Medicine, A-1124 Medical Center North, Nashville, TN 37232, USA

A number of recent epidemiological studies have pointed out that several characteristics of pregnancy and parturition are likely to be associated with the risk for breast cancer in the offspring. These characteristics include cerebral asymmetry that is believed to be conditioned by endocrine factors during the perinatal period (Sandson et al, 1992), birthweight which is likely to be affected by growth factors and perhaps exogenous influences (Michels et al, 1996; Sanderson et al, 1996; Ekbom et al, 1997), neonatal jaundice of unspecified aetiology (Ekbom et al, 1997) and, inversely, pregnancy toxaemia that is associated with profound but poorly understood endocrine changes (Ekbom et al, 1997). Although the dynamics of the intrauterine environment are clearly complex, hormones are central to its definition and notably oestrogens are necessary for the normal development of the breast. We have hypothesized that one or several of these hormones influence, already in utero, the risk of developing cancer during adult life. To evaluate this hypothesis, we have compared, for the first time, pregnancy hormone levels in samples from two populations with widely different rates for cancer of the breast – US and China.

### MATERIALS AND METHODS

### Subjects

Study subjects were adult pregnant women recruited from maternity clinics affiliated with two study centres, Beth Israel Hospital in Boston, MA, USA, and Shanghai Medical University in China. In Boston, women were urban residents, whereas in Shanghai they were recruited from three urban clinics and one rural clinic. In each centre, an authorized health professional met all pregnant women coming for their first routine prenatal visit to the collaborating maternity clinic, ascertained whether the woman was eligible to participate, explained to her the objectives of the study and the requirements for participation, and obtained informed consent.

To be eligible for participation, a pregnant woman had to be less than 40 years of age, had to have no more than one previous stillborn or liveborn child, had to be Caucasian in Boston and Chinese in Shanghai, and had to be able to understand and speak the local language. Women were excluded if they had taken any hormonal medication during the index pregnancy, if they had a prior diagnosis of diabetes mellitus or thyroid disease, or if the fetus had a known major anomaly.

Between March 1994 and October 1995, a total of 402 eligible women were identified at Beth Israel Hospital in Boston. Of these, 77 (19.2%) refused to participate, 11 (2.7%) were subsequently excluded because of early pregnancy termination, either spontaneous or induced ($n$ = 9), or twin birth ($n$ = 2), whereas ten (2.5%) were lost to follow-up after the initial meeting; thus, 304 pregnant women were eventually included from the Boston site. In Shanghai, a total of 424 eligible women were identified between April 1994 and May 1995. Of these, 15 (3.5%) refused to participate, ten (2.4%) were subsequently excluded because of induced abortion ($n$ = 2), twin birth ($n$ =2), or implied gestation duration <30 or >50 weeks ($n$ = 6), whereas 58 (13.7%) provided no blood samples and seven (1.7%) were lost to follow-up after the initial meeting; thus, 334 pregnant women were eventually enrolled in the study from the Shanghai site.

Hormone levels were measured in maternal blood. Maternal oestrogen levels have been shown to increase and correlate well with gestational age, so that two measurements during pregnancy will characterize better the mammary gland exposure to oestrogens and other hormones during gestation. Therefore, the first sampling was scheduled to take place around the 16th (completed) week of gestation. The second measurement was done around the 27th (completed) week of gestation; by then, the pregnancy is well advanced and the gestational age-dependent increase of oestradiol (E2) appears to slow down, thus minimizing the consequences of the unavoidable small variability in the timing of blood sampling.

In both study centres, gestational age was defined as the time since the first day of the last menstrual period. Around the 16th week visit, blood pressure and baseline demographic information were abstracted from the medical records of consenting participants and blood was drawn. Around the 27th week visit, the authorized health professional conducted a confidential interview with the study participant and abstracted relevant data from her records; again, blood was drawn. In addition, at each of the two visits, women were asked to complete a self-administered food frequency questionnaire pertaining to their dietary patterns during the previous trimester of pregnancy. At delivery, the placenta was weighed before discarding; additional information concerning the delivery and the newborn was ascertained from medical records and paediatric charts and recorded at the pregnancy's term.

### Blood collection

From every woman, at each visit, 10 ml of venous blood was drawn and collected into centrally provided sterile tubes without preservative. After centrifugation, the serum from each sampling was separated and equally distributed into aliquots centrally provided and labelled with a blood sample number which was linked, through the questionnaire, to subject's name, study visit and time and date of blood draw. All tubes had uniform labelling for both Boston and Shanghai samples and for both study visits.

For the Boston site and the urban hospitals in China, blood samples were kept refrigerated at 4°C for up to 24 h until centrifugation. For the rural hospital of the China site, blood samples were drawn between 8 a.m. and 10 a.m. and kept refrigerated at 4°C until they were transported in a cooler to a laboratory near Shanghai Medical University and centrifuged, between 1 p.m. and 2 p.m. the same day. The aliquots were stored at –20°C for about 5–7 days in the laboratory before being transported to Shanghai Medical University and stored at –80°C. The aliquots of blood from all women were stored locally at –80°C until all samples were collected. The aliquots from Shanghai were then packed in dry ice and transported to Boston in person by a researcher; the sample were stored at –80°C in Boston until being shipped by air, together with the Boston samples, to Uppsala, Sweden, where all biochemical analyses were undertaken by one of us (LW).

Specifically, the aliquots were packed in dry ice, adequate to last for at least twice the estimated length of transport time. It is unlikely that any thawing occurred before the transport of the serum samples to Uppsala; in any case, steroid hormones are quite stable even when samples are frozen at –20°C and are repeatedly thawed and refrozen (Kley and Rick, 1984; Kley et al, 1985; Sinnecker, 1989).

### Laboratory analyses

Sera which were clearly haemolysed and several with illegible labels were omitted from the laboratory analyses. An equal number of samples from each study site were analysed, the exclu-

 © Cancer Research Campaign 1999

sion of surpluses done through a strictly random process. In the assay series, every second sample was from Boston and Shanghai respectively. Thus, the number of serum specimens included from each site was 303 for the first sample collection and 297 for the second.

Four pools of serum samples served as controls: Shanghai first sample collection, Shanghai second collection, Boston first collection and Boston second collection. Specimens from each pool were placed at regular intervals in the series, treated like test samples, and measured a total of 24 times for each analyte (hormone). The mean levels of these control specimens, as well as the mean (±s.d.) imprecision for each of the seven analytes, were calculated.

Oestradiol-17β (E2) in 25 μl serum, diluted 1:20, was measured with a time-resolved competitive solid-phase fluoroimmunoassay (AutoDELFIA Estradiol kit; Wallac Oy, Turku, Finland), based on competition between europium-labelled oestradiol and sample oestradiol for polyclonal anti-oestradiol antibodies (derived from rabbit). A second antibody, directed against rabbit IgG, is coated onto the walls of microtitre plates, and binds the IgG–oestradiol complex. The cross-reactivity for oestrone and oestriol at the 50% inhibition level was 0.75% and 0.40% respectively. The results were expressed in nmol $l^{-1}$. The imprecision was 4.6±0.8%.

Unconjugated oestriol (E3) in 50 μl serum was measured with a similar time-resolved competitive solid-phase fluoroimmunoassay method (AutoDELFIA Unconjugated Estriol kit, Wallac Oy, Turku, Finland). The cross-reactivity at 50% inhibition was for oestriol-3-sulphate 37%, for oestriol-3-glucuronide 37% and for oestradiol <0.1%. The results were expressed in nmol $l^{-1}$. The imprecision was 8.0±1.8%.

Prolactin (hPRL) in 25 μl of serum was measured with a time-resolved non-competitive solid-phase sandwich fluoroimmunoassay in which two monoclonal antibodies (derived from mice) are directed against two separate antigenic determinants on the hPRL molecule (AutoDELFIA Prolactin kit; Wallac Oy, Turku, Finland). The results were expressed in μg $l^{-1}$. One μg = 36 mU of the WHO 3rd International Standard (84/500) for prolactin. The imprecision was 3.0±0.3%.

Progesterone in 25 μl serum, diluted 1:8, was measured with a similar time-resolved competitive solid-phase fluoroimmunoassay method (AutoDELFIA Progesterone kit; Wallac Oy, Turku, Finland). The results were expressed in nmol $l^{-1}$. The imprecison was 1.67±0.9%.

Human growth hormone (hGH) in 25 μl serum was measured with similar time-resolved non-competitive solid-phase sandwich fluoroimmunoassay (AutoDELFIA hGH, Wallac OY, Turku, Finland). The hGH assay was specific for the pituitary 22-kDa isoforms of hGH. The results were expressed in mU $l^{-1}$, using a recombinant human pituitary GH (22 kDa) as a standard calibrated against the WHO 1st International Reference Preparation (80/505) for hGH. The imprecision was 2.9±0.2%.

Albumin in 3 μl serum was measured with an automated analysis Hitachi System 717, using bromocresol green as a reagent. The method was calibrated against an immunoassay for human serum albumin. The results were expressed in g $l^{-1}$. The imprecision was 1.8±0.2%.

Sex hormone-binding globulin (SHBG) in 25 μl serum, diluted 1:100, was measured with similar time-resolved non-competitive solid-phase sandwich fluoroimmunoassay (AutoDELFIA SHBG kit, Wallac OY, Turku, Finland). The results were expressed in nmol $l^{-1}$, using as a reference a recombinant human SHBG preparation. The imprecision was 4.8±1.3%.

### Statistical analyses

Non-parametric Wilcoxon's rank-sum test was applied in univariate comparisons of the hormone levels between the two study sites (Armitage and Berry, 1987). Multivariate analysis was based on modelling through multiple regression, using the logarithm of the hormone levels as the dependent variables. The level of statistical significance was set at $P<0.05$, two-sided.

### RESULTS

Maternal, newborn and gestation characteristics are presented for American and Chinese women in Table 1. The age range of women at each study centre was 19–39 years. The mean age was higher among American (31 years) than among Chinese (24 years) women. Also, pregnant women in Boston were on average taller and heavier than pregnant women in Shanghai. With respect to newborn characteristics, those born to American women had higher birthweight than those born to Chinese women. On average, total duration of gestation was similar among the two groups, as were completed weeks of gestation at the first blood sampling stage; the second blood sample was, however, collected on average 1 day earlier in gestation among Chinese than in American women. With respect to parity status, 36.8% of American women reported a previous full-term pregnancy, compared with only 3% of Chinese women – an expected finding given the restrictions on family size in China.

Table 2 shows concentrations of the measured hormonal variables and of albumin and SHBG in serum samples collected at about 16 and 27 completed weeks of gestation among pregnant women in Boston and Shanghai. For each of the variables, values were generally within the expected range for pregnant women at those particular stages of pregnancy. Median levels of all the studied hormones were statistically significantly – and in most instances substantially – higher among Chinese women during the first visit ($P<0.01$). An analogous pattern was evident during the second visit, although the relative differences tended to be smaller and, for progesterone, the values were actually somewhat higher among American women. The magnitudes of the differences between Chinese and American women remained essentially unchanged after adjusting for maternal age and parity in a regression analysis that used the log-transformed hormone level as the dependent variable. There were no statistically significant differences among the four clinics in Shanghai (data not shown).

Minor differences by gender of the offspring were noted that reached statistical significance at the 0.05 level with respect to: progesterone levels among American women at both visits (median levels for women with male or female fetus were 139 and 125 nmol $l^{-1}$, respectively, at first visit, and 273 and 240 nmol $l^{-1}$, respectively, at second visit); oestradiol levels among Chinese women at second visit (median levels for women with male or female fetus were 42.8 and 46.2 nmol $l^{-1}$ respectively).

### DISCUSSION

This study was designed to address the question of whether hormonal factors during pregnancy could explain a series of associations reported from different populations between perinatal characteristics and breast cancer risk. In the process, data concerning hormone levels at two pregnancy stages for Caucasian women in the US and for Chinese women in Shanghai were

© Cancer Research Campaign 1999 

**Table 1** Maternal, newborn and gestation characteristics among 304 pregnant women from Boston, USA, and 334 pregnant women from Shanghai, China

| | | *n*[a] | Mean ± s.d. | Median | Range | *P*-values[b] |
|---|---|---|---|---|---|---|
| Maternal | | | | | | |
| Age (years) | Boston | 304 | 31.0 ± 3.1 | 31 | 19–39 | 0.0001 |
| | Shanghai | 334 | 25.1 ± 3.8 | 24 | 19–39 | |
| Weight (kg) | Boston | 296 | 60.0 ± 9.8 | 59 | 39–113 | 0.0001 |
| | Shanghai | 332 | 51.2 ± 6.0 | 50 | 35–75 | |
| Height (cm) | Boston | 298 | 164.3 ± 6.5 | 165 | 150–188 | 0.0001 |
| | Shanghai | 334 | 160.1 ± 4.7 | 160 | 147–174 | |
| BMI (kg $m^{-2}$) | Boston | 296 | 22.2 ± 3.2 | 21.5 | 16.8–36.0 | 0.0001 |
| | Shanghai | 332 | 20.0 ± 2.1 | 19.8 | 14.3–28.6 | |
| Newborn | | | | | | |
| Birthweight (g) | Boston | 304 | 3501 ± 538 | 3515 | 1725–4970 | 0.004 |
| | Shanghai | 334 | 3392 ± 463 | 3400 | 1800–4800[c] | |
| Birth length (cm) | Boston | 304 | 50.3 ± 2.6 | 51 | 37–57 | 0.01 |
| | Shanghai | 334 | 49.8 ± 2.7 | 50 | 30–56 | |
| Head circumference (cm) | Boston | 296 | 34.4 ± 1.8 | 34.5 | 24–38.5 | 0.84 |
| | Shanghai | 320 | 34.5 ± 2.0 | 34 | 28–41[d] | |
| Placenta weight (g) | Boston | 210 | 586.5 ± 154.1 | 580 | 280–1020[e] | 0.009 |
| | Shanghai | 302 | 610.4 ± 143.4 | 600 | 226–1000[f] | |
| Gestation | | | | | | |
| Total duration (weeks) | Boston | 294 | 39.9 ± 1.9 | 40 | 31.7–48.3 | 0.60 |
| | Shanghai | 326 | 39.8 ± 1.8 | 39.9 | 31.1–47.7 | |
| At sample 1 (weeks) | Boston | 278 | 16.8 ± 1.3 | 16.6 | 12.7–27.1 | 0.83 |
| | Shanghai | 283 | 16.9 ± 1.6 | 16.7 | 11.7–24.7 | |
| At sample 2 (weeks) | Boston | 276 | 27.2 ± 1.9 | 27.6 | 21.7–37.1 | 0.002 |
| | Shanghai | 275 | 27.0 ± 1.6 | 26.7 | 20.3–38.1 | |

[a]The differences reflect missing values. [b]Wilcoxon rank-sum test *P*-values comparing US and Chinese women. [c]One observation with birthweight = 998 g. [d]Two observations with head circumference = 51 cm. [e]One observation with placenta weight = 1620 g. [f]One observation with placenta weight = 1600 g.

**Table 2** Serum levels of studied hormones at 16 and 27 completed weeks of gestation among pregnant women in Boston, USA, and in Shanghai, China[a]

| | | Sample 1 | | | | Sample 2 | | | |
|---|---|---|---|---|---|---|---|---|---|
| Hormone | | *n* | Mean ± s.d. | Median | Range | *n* | Mean ± s.d. | Median | Range |
| Oestradiol (E2) | Boston | 288 | 14.0 ± 6.4 | 12.6 | 3.1–43.3 | 286 | 39.1 ± 16.6 | 36.4 | 12.9–145.9 |
| (nmol $l^{-1}$) | Shanghai | 291 | 20.7 ± 9.7 | 18.3 | 5.1–67.7 | 283 | 48.2 ± 18.3 | 44.2 | 16.7–152.9 |
| Oestriol (E3) | Boston | 288 | 3.9 ± 1.7 | 3.5 | 0.4–11.0 | 286 | 14.0 ± 4.4 | 13.5 | 5.8–30.5 |
| (nmol $l^{-1}$) | Shanghai | 291 | 6.3 ± 3.9 | 5.4 | 1.03–27.8 | 283 | 21.9 ± 9.5 | 20.4 | 7.6–107.0 |
| Prolactin | Boston | 288 | 44.7 ± 24.5 | 40.2 | 5.5–210.5 | 286 | 91.3 ± 35.5 | 89.4 | 19.4–321.4 |
| (µg $l^{-1}$) | Shanghai | 291 | 63.1 ± 32.5 | 58.8 | 12.5–274.1 | 283 | 116.0 ± 37.5 | 113.3 | 41.8–266.9 |
| Progesterone | Boston | 288 | 133.0 ± 31.8 | 131.0 | 57.3–255.0 | 286 | 263.3 ± 69.3 | 252.0 | 109.0–544.0 |
| (nmol $l^{-1}$) | Shanghai | 291 | 143.3 ± 35.9 | 139.0 | 67.6–305.0 | 283 | 247.8 ± 80.5 | 236.0 | 112.0–735.0 |
| Growth hormone | Boston | 288 | 2.98 ± 3.84 | 1.43 | 0.02–22.94 | 286 | 0.90 ± 1.54 | 0.28 | 0.02–11.0 |
| (mU $l^{-1}$) | Shanghai | 291 | 3.69 ± 3.78 | 2.35 | 0.05–19.46 | 283 | 1.74 ± 2.55 | 0.80 | 0.05–25.4 |
| Albumin | Boston | 288 | 40.2 ± 2.2 | 40.3 | 34.5–47.1 | 286 | 36.5 ± 2.0 | 36.6 | 30.9–42.8 |
| (g $l^{-1}$) | Shanghai | 291 | 43.2 ± 2.6 | 43.1 | 36.4–50.3 | 283 | 39.6 ± 2.2 | 39.3 | 34.4–46.3 |
| SHBG | Boston | 288 | 362.4 ± 90.4 | 365.9 | 117.9–606.3 | 286 | 425.6 ± 111.2 | 420.2 | 137.9–774.5 |
| (nmol $l^{-1}$) | Shanghai | 291 | 429.4 ± 90.8 | 427.1 | 205.0–653.9 | 283 | 479.5 ± 122.3 | 465.8 | 186.1–977.6 |

[a]All differences between sites at each visit were statistically significant ($P$<0.05).

generated; these data could be of general use in reproductive and perinatal epidemiology.

Early life events and conditions, including the intrauterine period, have recently received increasing attention in the context of several adult-onset diseases, including cardiovascular diseases (Bakketeig et al, 1991; Barker, 1995) and a number of malignancies (Kaye et al, 1991; Cnattingius et al, 1995; Tibblin et al, 1995; Akre et al, 1996; Michels et al, 1996; Sanderson et al, 1996; Ekbom et al, 1996, 1997). Among adult-onset cancers, the data are stronger with respect to breast cancer: the evidence comes from studies showing a positive association of birth size indicators with both breast cancer itself and high-risk mammograms (Ekbom et al, 1995, 1997;

 *© Cancer Research Campaign 1999*

Michels et al, 1996; Sanderson et al, 1996), findings which suggest that the excess risk for breast cancer is higher when a sister rather than the mother is affected among first-degree relatives (Eby et al, 1994; Bernstein et al, 1995; Hunter et al, 1997), investigations linking cerebral asymmetry to breast cancer risk (Sandson et al, 1992), and data indicating that pregnancy toxaemia is inversely and neonatal jaundice is positively associated with breast cancer risk in the offspring (Ekbom et al, 1997).

Of the steroid hormones measured in the present study, progesterone is entirely of indirect maternal origin, formed by the placenta from maternal low density lipoprotein (LDL) cholesterol. Oestradiol-17β is synthesized in the placenta by 50% from maternal and by 50% from fetal androgen precursors. In contrast, oestriol is formed by more than 90% by placental conversion of fetal 16α-hydroxydehydroepiandrosterone and its sulphate (Falcone and Little, 1994). Oestradiol-17β and, especially, oestriol levels were considerably higher in Chinese than in American women at both sampling stages. This difference would still remain after adjustment for different placental weight/bodyweight ratios, and for a relative dehydration in the Chinese women as indicated by their higher serum albumin values. The elevated oestrogen levels in Chinese women may, thus, reflect an increased production by these women of androgens from the fetal adrenal glands, including 16α-hydroxydehydroepiandrosterone and dehydroepiandrosterone. Little is known about the effects of these steroids on breast development. However, an increased adrenal androgen production may reflect differences in other factors, such as growth factors, that could affect breast development (Murphy and Branchaud, 1994).

Because oestrogens in the adult life, whether endogenous or exogeneous (Peto, 1989; Toniolo et al, 1995), appear to be important in breast carcinogenesis, it would be natural to speculate that high levels of pregnancy oestrogens would be critical for the intrauterine origin of this disease. The data presented here, however, contradict strongly a straightforward positive association between actual oestrogen levels during pregnancy and risk for breast cancer – whether one focuses on oestradiol (E2) or the abundantly produced oestriol (E3). In fact, in the present study, all measured pregnancy hormones as well as SHBG and albumin were substantially higher among Chinese women than among Caucasian American women, with the possible exception of progesterone during the late stage sampling. This ecological contrast contradicts the a priori hypothesis that invokes levels of pregnancy oestrogens as an important factor in the intrauterine processes that modulate breast cancer risk. Oestrogens and other pregnancy hormones, however, could influence breast cancer risk through more indirect or complex processes.

There are various plausible mechanisms that could accommodate an oestrogen-mediated effect on breast cancer risk as well as higher levels of pregnancy oestrogens in the low-risk population. First, it is possible that higher oestrogen levels in the intrauterine period down-regulate the expression of oestrogen receptors in breast tissue throughout life through some form of imprinting. There is little information about concentrations of oestrogen receptors in normal breast tissue compared with breast cancer, but what information exists does indicate that concentrations are substantially higher in Caucasian women than in Japanese women, who share with Chinese a low risk for breast cancer (Punnonen et al, 1984; Pegoraro et al, 1986). Second, higher levels of pregnancy oestrogens in conjunction with high levels of other mammotrophic hormones could advance differentiation of mammary cells, making them less susceptible to carcinogenesis, in the context of a mechanism postulated by Russo and Russo (1996). Last, it is possible that the higher albumin and SHBG levels among Chinese could imply different bioavailability of oestrogens in Chinese compared with American women, although lack of data on other steroids, particularly testosterone, and perhaps other relevant variables in the intrauterine hormonal environment hinders estimation of free oestradiol. As a corollary in adult life, breast cancer patients have (even before diagnosis) consistently lower levels of SHBG (Lipworth et al, 1996).

The data of the present study confirm that birthweight is lower among Chinese than among Caucasian women, even though none of the measured growth factors could explain the difference. It has been suggested that the intercorrelated size of the stem cell pool (Albanes and Winick, 1988; Trichopoulos and Lipman, 1992; Trichopoulos and Lipworth, 1995) or number of replications (Albanes and Winick, 1988; Preston-Martin et al, 1990) could be a background risk factor on which the effects of later-acting causes of breast cancer are superimposed.

The evidence linking perinatal factors to breast cancer risk is strong (Adami et al, 1995; Michels et al, 1996; Ekbom et al, 1997). The present study demonstrates striking differences in the intrauterine endocrine environment between American and Chinese women. This large amount of data concerning pregnancy hormones and their correlates should pave the way for a better understanding of the physiological processes during gestation and the way these could affect perinatal and adult life events and conditions. Although we have discussed our findings in relation to breast cancer, they clearly could be relevant to other malignancies, such as cancer of the testis and prostate (Akre et al, 1996; Ekbom et al, 1996), the incidence of which differs between the two studied populations.

### ACKNOWLEDGEMENTS

This study was supported in part by grant no. CA54220 from the National Institutes of Health. The authors would like to acknowledge Joanne Wuu for assisting with the statistical analyses, Janet Spencer, Kristin Daley, Pat Morey and Naomi Lieberman for conducting patient interviews and abstracting medical records, Xu-Liang Li, Shi-Lin Ren and Pai-Fang Chin for conducting field work, and Dimitri Seretakis for assisting with manuscript preparation. We are grateful to the following physicians for their participation: at Beth Israel Hospital in Boston, Benjamin Sacks, Ruth Fretts, Eric Lichter, Roxanne Gardner, Laura Bookman, Johanna Perlmutter, Mark Kaplan, Ronald Marcus, Hope Ricciotti, David Hagen, Todd Shapiro, Lenore Soodak, Ralph Aserkoff, and Jennifer Shaw; at Shanghai Nanhui County Zhoupu Hospital, Ming-Gen Miao, Wen-Juan Cai, Ming-Rong Tang, Feng-Ming Gui, Ai-Juan Qu, and Bi-Hua Xu; at Shanghai No. 2 People's Hospital, Jin-Shen Fa, Weng-Juan Fang, Li-Fang Shen; at Shanghai International Peace Maternal and Child Hospital, Yue-Hua Shen, Zhi-Yin Zhen, Rong-Zhen Fi, Xue-Yi Zhang, Ziu-Yin Xia, Hui-Ping Chen, Qin-Ping Zhou, Yue-Fang Shen, and Meng-Kun Lu; at Shanghai Medical University Affiliated Obstetrics and Gynecology Hospital, Ming-ming Pan and Xiao-Min Hu.

### REFERENCES

Adami H-O, Adams G, Boyle P, Ewertz M, Lee NC, Lund E, Miller AB, Olsson H, Steel M, Trichopoulos D and Tulinius H (1990) Breast cancer etiology. *Int J Cancer* (suppl) **5**: 22

*© Cancer Research Campaign 1999*

Adami H-O, Persson I, Ekbom A, Wolk A, Ponten J and Trichopoulos D (1995) The aetiology and pathogenesis of human breast cancer. *Mutat Res* **333**: 29–35
Akre O, Ekbom A, Hsieh C-C, Trichopoulos D and Adami H-O (1996) Testicular nonseminoma and seminoma in relation to perinatal characteristics. *J Natl Cancer Inst* **88**: 883–889
Albanes D and Winick M (1988) Are cell number and cell proliferation risk factors for cancer? *J Natl Cancer Inst* **80**: 772–775
Armitage P and Berry G (1987) *Statistical Methods in Medical Research*. Blackwell: Boston
Bakketeig LS, Magnus P and Sundet JM (1991) Differential development of health in a life-span perspective. In *Problems and Methods in Longitudinal Research: Stability and Change*. Magnusson D, Bergman LR, Rudinger G and Torestad B (eds), pp. 95–106. Cambridge University Press: Cambridge
Barker DJP (1995) Fetal origins of coronary heart disease. *Br Med J* **311**: 171–174
Bernstein L, Lipworth L, Ross RK and Trichopoulos D (1995) Correlation of estrogen levels between successive pregnancies. *Am J Epidemiol* **142**: 625–628
Cnattingius S, Zack MM, Ekbom A, Gunnarskog J, Kreuger A, Linet M and Adami H-O (1995) Prenatal and neonatal risk factors for childhood lymphatic leukemia. *J Natl Cancer Inst* **87**: 908–914
Cole P and MacMahon B (1969) Oestrogen fractions during early reproductive life in the aetiology of breast cancer. *Lancet* **1**: 604–606
deWaard F (1975) Breast cancer incidence and nutritional status with particular reference to body weight and weight. *Cancer Res* **35**: 3351–3356
Eby N, Chang-Claude J and Bishop DT (1994) Familial risk and genetic susceptibility for breast cancer. *Cancer Causes Control* **5**: 458–470
Ekbom A, Thurfjell E, Hsieh C-C, Trichopoulos D and Adami H-O (1995) Perinatal characteristics and adult mammographic patterns. *Int J Cancer* **61**: 177
Ekbom A, Hsieh C-C, Lipworth L, Wolk A, Ponten J, Adami H-O and Trichopoulos D (1996) Perinatal characteristics in relation to incidence of and mortality from prostate cancer. *Br Med J* **313**: 337–341
Ekbom A, Hsieh C-C, Lipworth L, Adami H-O and Trichopoulos D (1997) Intrauterine environment and breast cancer risk in women: a population-based study. *J Natl Cancer Inst* **88**: 71–76
Falcone T and Little AB (1994). Placental synthesis of steroid hormones. In *Maternal–Fetal Endocrinology*. Tulchinsky D and Little AB (eds), pp. 1–14. Saunders: Philadelphia
Hsieh C-C, Trichopoulos D, Katsouyanni K and Yuasa S (1990) Age at menarche, age at menopause, height and obesity as risk factors for breast cancer: associations and interactions in an international case–control study. *Int J Cancer* **46**: 796–800
Hunter DJ, Spiegelman D, Adami HO, Beeson L, van den Brandt PA, Folsom AR, Fraser GE, Goldbohm RA, Graham S, Howe GR, Kushi LH, Marshall JR, McDermott A, Miller AB, Speizer FE, Wolk A, Yuan S-S and Willett W (1996). Cohort studies of fat intake and the risk of breast cancer – a pooled analysis. *N Engl J Med* **334**: 356–361
Hunter DJ, Spiegelman D, Adami H-O, van den Brandt PA, Folsom AR, Goldbohm RA, Graham S, Howe GR, Kushi LH, Marshall JR, Miller AB, Speizer FE, Willett W, Wolk A and Yuan S-S (1997) Non-dietary factors as risk factors for breast cancer, and as effect modifiers of the association of fat intake and risk of breast cancer. *Cancer Causes Control* **8**: 49–56
Kaye SA, Robison LL, Smithson WA, Gunderson P, King FL and Neglia JP (1991) Maternal reproductive history and birth characteristics in childhood acute lymphoblastic leukemia. *Cancer* **68**: 1351–1355
Kley HK and Rick W (1984) Einfluβ von lagerung und temperatu auf die analyse von steroiden in plasma und blud. *J Clin Chem Clin Biochem* **22**: 371–378
Kley HK, Schlaghecke R and Kruskemper HL (1985) Stabilitat von steroiden im plasma uber einen zeitraum von 10 jahren. *J Clin Chem Clin Biochem* **23**: 875–878
Lipworth L (1995) Epidemiology of breast cancer. *Eur J Cancer Prev* **4**: 7–30
Lipworth L, Adami H-O, Trichopoulos D, Carlström K and Mantzoros C. (1996) Serum steroid hormones, sex hormone-binding globulin and body mass index in the etiology of postmenopausal breast cancer. *Epidemiology* **7**: 96–100
Michels KB, Trichopoulos D, Robins JM, Rosner BA, Manson JE, Hunter DJ, Colditz GA, Hankinson SE, Speizer FE and Willett WC (1996) Birthweight as a risk factor for breast cancer. *Lancet* **348**: 1542–1546
Murphy BEP and Branchaud CL (1994) The fetal adrenal. In *Maternal–Fetal Endocrinology*. Tulchinsky D and Little AB (eds), pp. 275–295. Saunders: Philadelphia
Pegoraro RJ, Nirmul D, Reinach SG, Jordaan JP and Joubert SM (1986) Breast cancer prognosis in three different racial groups in relation to steroid hormone receptor status. *Breast Cancer Res Treatment* **7**: 111–118
Peto J (1989) Oral contraceptives and breast cancer: is the CASH study really negative? *Lancet* **1**: 552
Pike MC, Krailo MD, Henderson BE, Casagrande TJ and Hoel DG (1983) ‘Hormonal’ risk factors, ‘breast tissue age’, and the age-incidence of breast cancer. *Nature* **303**: 767–770
Preston-Martin S, Pike MC, Ross RK, Jones PA and Henderson BE (1990) Increased cell division as a cause of human cancer. *Cancer Res* **50**: 7415–7421
Punnonen R, Lukola A and Kudo R (1984) Cytoplasmic estrogen receptor concentrations in the endometrium of Finnish and Japanese women. *Eur J Obstet Gynecol Reprod Biol* **17**: 321–325
Rogers AE and Longnecker MP (1988) Dietary and nutritional influences on cancer: a review of epidemiologic and experimental data. *Laboratory Invest* **59**: 729–759
Russo IH and Russo J (1996) Mammary gland neoplasia in long-term rodent studies. *Environ Health Perspect* **104**: 938–967
Sanderson M, Williams MA, Malone KE, Stanford JL, Emanuel I, White E and Daling JR (1996) Perinatal factors and risk of breast cancer. *Epidemiology* **7**: 34–37
Sandson TA, Wen PY and LeMay M (1992) Reversed cerebral asymmetry in women with breast cancer. *Lancet* **339**: 523–524
Sinnecker G (1989) Stability of sex-hormone-binding globulin in serum and plasma. *Clin Chem* **35**: 1253–1254
Tibblin G, Eriksson M, Cnattingius S and Ekbom A (1995) High birthweight as a predictor of prostate cancer risk. *Epidemiology* **6**: 423–424
Tomatis L (1989) Overview of perinatal and multi-generation carcinogenesis. In *Perinatal and Multigeneration Carcinogenesis*, Napalkov NP, Rice JM, Tomatis L and Yamasaki H (eds), Vol. 96. pp. 1–15. IARC: Lyon
Toniolo PG, Levitz M, Zeleniuch-Jacquotte A, Banerjee S, Koenig KL, Shore RE, Strax P and Pasternack BS (1995) A prospective study of endogenous estrogens and breast cancer in postmenopausal women. *J Natl Cancer Inst* **87**: 190–197
Tretli S (1989) Height and weight in relation to breast cancer morbidity and mortality. A prospective study of 570,000 women in Norway. *Int J Cancer* **44**: 23–30
Trichopoulos D and Lipman RD (1992) Mammary gland mass and breast cancer risk. *Epidemiology* **3**: 523–526
Trichopoulos D and Lipworth L (1995) Is cancer causation simpler than we thought, but more intractable? *Epidemiology* **6**: 347–349
Valaoras V, MacMahon B, Trichopoulos D and Polychronopoulou A (1969) Lactation and reproductive histories of breast cancer patients in Greater Athens, 1965–67. *Int J Cancer* **4**: 350–363
van den Brandt PA, Dirx MJM, Ronckers CM, van den Hoogen P and Goldbohm RA (1997) Height, weight, weight change, and postmenopausal breast cancer risk: the Netherlands Cohort Study. *Cancer Causes Control* **8**: 39–47
Ziegler RG, Hoover RN, Pike MC, Hildesheim A, Nomura AMY, West DW, Wu-Williams AH, Kolonel LN, Horn-Ross PL, Rosenthal JF and Hyer MB (1993) Migration patterns and breast cancer risk in Asian–American women. *J Natl Cancer Inst* **85**: 1819–1827

 © Cancer Research Campaign 1999
